# Visceral adiposity and renal function: an observational study from SPECT-China

**DOI:** 10.1186/s12944-017-0597-0

**Published:** 2017-10-27

**Authors:** Kun Zhang, Qin Li, Yi Chen, Ningjian Wang, Yingli Lu

**Affiliations:** Institute and Department of Endocrinology and Metabolism, Shanghai Ninth People’s Hospital, Shanghai Jiao Tong University School of Medicine, No.639 Zhizaoju Road, Shanghai, 200011 China

**Keywords:** Visceral adiposity dysfunction, Declined renal function, LAP index

## Abstract

**Background:**

Lipid accumulation product (LAP) is a novel and effective index of visceral adiposity distribution based on waist circumference and triglycerides concentration. Few studies investigated the relationship between LAP and eGFR. We aimed to explore whether LAP was associated with declined renal function (eGFR < 60 mL/min/1.73m^2^), and also whether it exhibited obvious superiority in predicting kidney impairment compared with other obesity indices.

**Methods:**

In this cross-sectional study, 10,012 subjects were recruited from 22 sites in East China. LAP was calculated with the following formula: (WC-65) x TG for males, (WC-58) x TG for females.

**Results:**

4.7% participants with declined renal function had a higher LAP quartile. LAP was strongly associated with eGFR level (Beta: -0.073, *P* < 0.001) and declined renal function (P < 0.001) even after adjustment for age, sex, smoking, drinking, diabetes and hypertension. The risk of renal dysfunction increased 2.32-fold for the highest quartile LAP relative to the lowest quartile (OR: 2.32, 95%CI:1.52–3.53, *P* < 0.001). LAP exerted the largest area under the curve among different obesity indices (AUC ROC:0.644, 95%CI: 0.618–0.670, P < 0.001).

**Conclusions:**

Our findings showed that LAP strongly associated with declined renal function and could be one of markers for predicting the risk of renal dysfunction in the general Chinese population.

**Trial registration:**

ChiCTR-ECS-14005052 (WHO international clinical trials register platform in China). Registered 20 July 2014.

## Background

Chronic kidney disease (CKD) has attracted extensive attention during the past decades as a major challenge for global health. The CKD prevalence rate in the general Chinese population has reached 10.8% in 2012 [1]. Rapid increase in the CKD prevalence rate will have negative influences on China, including a large economic burden and poor quality of life [2–4]. Early identification and management of CKD is therefore beneficial to control and delay the progression to end-stage renal disease (ESRD) and other cardiovascular events.

Extensive epidemiological studies have indicated that obesity, especially visceral obesity, is involved in the development of chronic kidney disease [5, 6] and that an obese phenotype, independent of diabetes and hypertension, predicts a higher risk of CKD [7]. Traditional obesity index of BMI has been widely used in clinical practice, but failed to distinguish lean body mass from adipose tissue limits its application in the assessment of adiposity distribution [8]. Waist circumference (WC) is a more frequently used index of visceral obesity [9]. However, whether waist circumference is associated with declined kidney function is still controversial [10, 11]. Thus, we sought an appropriate indicator to evaluate the adverse influence of visceral obesity on kidney dysfunction. LAP, as a novel and effective metabolic index, showed promising potential in reflecting visceral adiposity dysfunction. LAP was first proposed by Kahn [12] to predict cardiovascular risk in 2005, which was determined by WC and triglycerides (TG) concentration. Recently, LAP was reported to have strong associations with insulin resistance [13, 14], diabetes [15], NAFLD [16] and even metabolic syndrome [17], all of which were associated with CKD [18]. However, there are few studies investigating the relationship between LAP and CKD in the general Chinese population. In addition, despite the significant influences of diabetes and hypertension on renal function, few studies have adjusted for these factors.

Based on the Survey on Prevalence in East China for Metabolic Diseases and Risk Factors (SPECT-China) conducted in 22 sites (Shanghai, Zhejiang, Anhui, Jiangsu and Jiangxi Province) between 2014 and 2015, we aimed to investigate whether LAP is associated with a decline in glomerular filtration rate (eGFR) in East China and then to further explore its ability to predict declined renal function.

## Methods

### Participants

Data are extracted from SPECT-China, a population-based, cross-sectional survey conducted to investigate the prevalence of metabolic disease and risk factors in East China (Registration number: ChiCTR-ECS-14005052, www.chictr.org.cn). A stratified cluster sampling method was performed, as previously reported [19]. Chinese citizens aged over 18 years who had lived at their current residence for 6 months or longer were selected to participate in the study. Those with acute illness, severe communication problems, or who were unwilling to participate were excluded. From January 2014 to December 2015, 10,441 adult subjects were recruited in the SPECT-China study from 22 sites in Shanghai, Zhejiang, Anhui, Jiangsu and Jiangxi Province. We excluded participants with missing values for BMI (*n* = 257) or WC (*n* = 167) or lipid profile (*n* = 4) or eGFR (n = 1). Finally, a total number of 10,012 participants ultimately were included in the study to investigate the association between declined renal function and LAP. Informed consent was obtained from all subjects before data collection. The study protocol was approved by the Ethics Committee of Shanghai Ninth People’s Hospital, Shanghai JiaoTong University School of Medicine. All procedures followed were in accordance with the ethical standards of the responsible committee on human experimentation (institutional and national) and with the Helsinki Declaration of 1975, as revised in 2008.

### Measurements

Data collection including information about demographic characteristics, medical history and lifestyle risk factors were completed by the same trained physicians and students at every survey site. Height and body weight were measured with subjects wearing light clothing and no shoes. BMI was defined as weight in kilograms divided by height in meters squared. Blood pressure was measured by sphygmomanometer following standard methods as previously described [20]. Neck circumference was performed with the following requirements. Subjects stood upright with their head placed in the Frankfort horizontal plane. The superior edge of the tape measure was placed just below the laryngeal prominence and applied perpendicular to the long axis of the neck. WC was measured midway between the inferior border of the last rib and the crest of the ilium in a horizontal plane. Hip circumference was measured around the pelvis at the point of the maximal protrusion of the buttocks. Smoking was described as having smoked at least 100 cigarettes in one’s lifetime and currently smoking cigarettes.

Blood samples were drawn from participants after an overnight fast of 8 h or longer. Plasma glucose and lipid profiles including fasting plasma glucose, TG, total cholesterol (TC), high density lipoprotein (HDL) and low density lipoprotein (LDL) were measured by BECKMAN COULTER AU 680 (Germany). HbA1c was measured by high-performance liquid chromatography (MQ-2000PT, China).

### Definition and calculation

eGFR was calculated according to the Chronic Kidney Disease-Epidemiology Collaboration (CKD-EPI) equation. Declined renal function was defined as eGFR less than 60 mL/min per1.73 m^2^. LAP was calculated by the following formula where WC was expressed in centimeter, TG in mmol/L: (WC-65) x TG for males, (WC-58) x TG for females. Visceral adiposity index (VAI) was calculated according to the formula: (WC/(39.68 + (1.88xBMI))) x (TG/1.03) x (1.31/HDL). Body adiposity index (BAI) was calculated as hip circumference in centimeter divided by height in meter-18. Diabetes was defined as a fasting plasma glucose of 7 mmol/L or higher, HbA1c of 6.5% or higher, or a previous diagnosis of type 2 diabetes. Hypertension was defined as systolic blood pressure ≥ 140 mmHg, diastolic blood pressure ≥ 90 mmHg, current use of antihypertensive drug, or self-reported history of hypertension.

### Statistical analysis

Statistical analyses were conducted by using IBM SPSS software, Version 23 (IBM Corporation, Armonk, NY, USA). General characteristics are described as the mean ± standard deviation (SD) for continuous variables or as a proportion (%) for categorical variables. To compare characteristics of the participants by LAP quartiles, the one-way ANOVA test was used for continuous variables, and a Chi-square test was used for categorical variables.

The LAP was divided into quartiles. The fourth quartile meant the highest and the first quartile displayed the lowest. The prevalence of declined renal function was calculated in each quartile. We used linear regression analyses to evaluate the association of eGFR with LAP and other obesity indices (BMI, VAI, WC and BAI) in three models. Model 1 was unadjusted. Model 2 controls for age, sex, smoking and drinking. Model 3 additionally controls for diabetes and hypertension. LAP and VAI were further log-transformed because of a high skew. Logistic regression was used to investigate the relationship between LAP and declined renal function. The first quartile of LAP served as the reference point. Data were expressed as odds ratios (OR) and 95% confidence intervals (CI). Adjusted models were the same as those in the linear regression. All of the analyses were two-sided. *P* < 0.05 was considered statistically significant.

To investigated the predictive abilities of LAP and other obesity indices for declined renal function, we compared the area under the curve (AUC) of the receiver operating characteristic (ROC) [21]. The differences of AUC between different indices and LAP were compared by Z-tests.

## Results

### Characteristics of the participants by quartiles of LAP quartiles

As presented in Table 1, the LAP quartile ranges were ≤13.03, 13.04–24.96, 24.97–45.62, ≥ 45.63, respectively. According to trend analysis, age, BMI, WC, neck circumference, hip circumference, BAI, TG, TC, LDL as well as the prevalence of hypertension and diabetes showed a dose-response relationship with LAP (all P for trend < 0.001). As expected, eGFR and HDL were negatively associated with LAP (all P for trend <0.001). Interestingly, this similar association was also observed in the prevalence of smoking and drinking (all P for trend <0.001).

**Table 1:** Characteristics of study population by quartiles of LAP

LAP value	Q1	Q2	Q3	Q4	*P* for trend
	≤13.03	13.04–24.96	24.97–45.62	≥45.63	
Male, %	36.4	40.5	41.0	46.8	<0.001
Age, year	47.38 (13.95)	52.98(12.56)	56.41 (11.86)	56.61 (11.56)	<0.001
Body mass index, kg/m^2^	21.45 (2.48)	23.79 (2.44)	25.41 (2.81)	27.43 (3.24)	<0.001
Waist circumference, cm	69.35 (5.76)	78.29 (6.08)	84.08 (6.81)	90.52 (8.18)	<0.001
Neck circumference, cm	31.61 (2.98)	33.06 (2.91)	34.08 (3.86)	35.54 (3.57)	<0.001
Hip circumference, cm	87.97 (5.21)	92.39 (5.37)	95.05 (6.09)	98.39(6.85)	<0.001
eGFR, mL/min/1.73 m^2^	94.63 (14.99)	89. 88 (14.59)	86.03 (14.91)	85.22 (15.16)	<0.001
Body adiposity index	25.28 (3.60)	27.32(3.64)	28.72 (4.20)	29.91 (4.86)	<0.001
Triglycerides, mmol/L	0.88 (0.32)	1.16 (0.41)	1.58 (0.53)	3.07 (2.53)	<0.001
Cholesterol, mmol/L	4.75 (1.06)	4.98 (1.04)	5.30 (1.04)	5.59 (1.17)	<0.001
HDL, mmol/L	1.59 (0.32)	1.46 (0.30)	1.39 (0.30)	1.27 (0.27)	<0.001
LDL, mmol/L	2.69 (0.64)	2.98 (0.70)	3.27 (0.77)	3.40 (0.81)	<0.001
Hypertension, %	23.9	40.0	54.4	66.4	<0.001
Diabetes, %	4.9	8.9	13.8	26.3	<0.001
Smoker, %	82.7	80.2	78.4	73.8	<0.001
Drinker, %	91.2	87.1	85.4	84.0	<0.001

Continuous variables are presented as the mean (standard deviation), and categorical variables are expressed as a proportion (%). *P* for trend was calculated by ANOVA and Chi-square test

### Prevalence of declined renal function

**Fig. 1:**
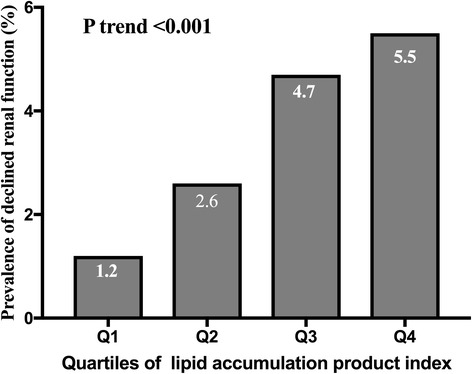
The prevalence of declined renal function in each quartile of LAP

Figure 1. showed the prevalence of declined renal function in each LAP quartile. They were 1.2%, 2.6%, 4.7% and 5.5%, respectively. As the LAP quartile increased, the prevalence of declined renal function increased, which implied the obvious association of LAP with the prevalence of declined renal function (P for trend <0.001).

### Association of LAP and other obesity indices with eGFR

Table 2. showed the results of the linear regression analysis investigating the association of different obesity indices with eGFR. In the model adjusted for no factors, log-LAP (Beta = −0.217), BMI (Beta = −0.152), waist circumference (Beta = −0.213), BAI (Beta = −0.147) and log-VAI (Beta = −0.136) were negatively associated with eGFR (all *P* < 0.001). After adjustment for age, sex, drinking and smoking, the association of log-LAP, BMI, WC and log-VAI with eGFR weakened but it was still significant (all *P* < 0.05). When additional adjustment for diabetes and hypertension, this association did not change but failed to further weaken (all P < 0.05).

**Table 2:** Association of eGFR with different obesity indices

Independent variable	eGFR (Dependent variable)		
	Model 1	Model 2	Model 3
Log-LAP	−0.217(0.157)**	−0.067(0.138)**	−0.073(0.144)**
BMI	−0.152(0.043)**	−0.071(0.036)**	−0.075(0.038)**
WC	−0.213(0.015)**	−0.030(0.014)*	−0.033(0.014)*
BAI	−0.147(0.034)**	−0.007(0.033)	−0.006(0.034)
Log-VAI	−0.136(0.512)**	−0.064(0.429)**	−0.069(0.442)**

The data from linear regression analysis are expressed as Beta coefficients (standard errors). *P < 0.05, **P < 0.001. Model 1 was unadjusted. Model 2 controls for age, sex, smoking and drinking. Model 3 additionally controls for diabetes and hypertension. VAI and LAP were log-transformed because of their skewed distribution

### Association of LAP with declined renal function

As presented in Table 3, the results of logistic regression analysis showed that the OR for declined renal function gradually increased across LAP quartiles in each model (all *P* < 0.001). In the unadjusted model (Model 1), the OR of declined renal function in the highest LAP was 4.62 (95% CI 3.12, 6.85; P < 0.001) compared with the lowest one. After adjustment for age, sex, drinking and smoking, LAP was still significantly associated with declined renal function but this interaction weakened (Model 2). With additional adjustment for diabetes and hypertension, this association did not change and the OR of declined renal function further deceased (Model 3).

**Table 3:** Association of LAP with declined renal function (eGFR <60 mL/min/1.73 m^2^)

LAP value	Model 1	Model 2	Model 3
Q1 (≤13.03)	1.0(ref.)	1.0(ref.)	1.0(ref.)
Q2 (13.04–24.96)	2.09(1.36, 3.22)	1.50(0.96,2.34)	1.45(0.93,2.27)
Q3 (24.97–45.62)	3.95(2.65,5.89)	2.20(1.45,3.33)	2.04(1.34,3.09)
Q4 (≥45.63)	4.62(3.12,6.85)	2.61(1.74,3.93)	2.32(1.52, 3.53)
*P* value for trend	<0.001	<0.001	<0.001

The data from logistic regression analysis are expressed as odds ratio (95% CI) unless otherwise indicated. Model 1 was unadjusted, Model 2 controls for age (continuous variable), sex, smoking, and drinking. Model 3 additionally controls for diabetes and hypertension

### AUCs of LAP and other obesity indices to predict declined renal function

**Fig. 2:**
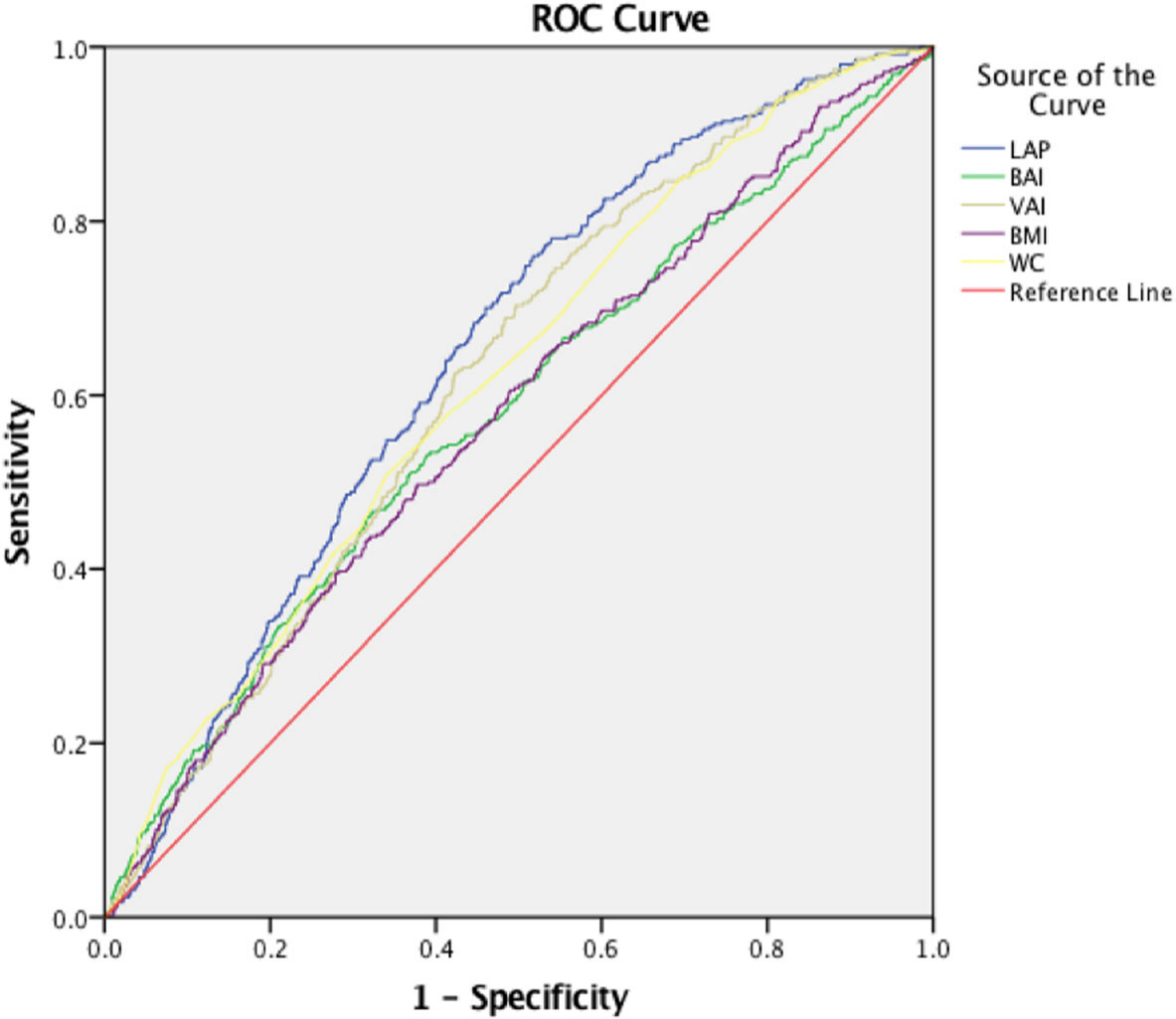
The association between LAP and declined renal function. In our study, we found that LAP was strongly associated with declined renal function. LAP has the largest area under curves among these five obesity indices, suggesting LAP can be a promosing predictive index for declined renal function

As presented in Table 4, we investigated the predictive abilities of LAP and other obesity indices for declined renal function. The AUC value of LAP was 0.644 (95% CI: 0.618–0.670). VAI, BAI, WC and BMI were inferior to LAP in AUC value. The BMI showed the lowest AUC value for declined renal function (Fig. 2).

**Table 4:** AUCs of LAP and other obesity indices to predict declined renal function

Index	AUC ROC	*P* value1	*P* value2
LAP	0.644 (0.618, 0.670)	<0.001	
VAI	0.621 (0.594, 0.647)	<0.001	<0.001
BAI	0.578 (0.546, 0.609)	<0.001	<0.001
BMI	0.574 (0.543, 0.605)	<0.001	<0.001
WC	0.616 (0.588, 0.645)	<0.001	<0.001

*AUC* area under the curve, *ROC* receiver operating characteristic. *P* value 1: the diagnostic value for ROC, two tail significance. *P* value 2: the comparisons of AUC between LAP and other obesity indices (Z-test)

## Discussion

In this large cross-sectional study, we explored the association between LAP and declined renal function in East China. We found that LAP, like other obesity indices, was strongly associated with eGFR level and declined renal function even after adjustment for age, sex, smoking, drinking, diabetes and hypertension. The full-adjusted risk of declined renal function grew by 2.32 times for the highest LAP quartile relative to the lowest quartile. LAP showed a more promising predictive ability for declined renal function compared to other obesity indices. To the best of our knowledge, this is the first large population-based study to directly compare LAP and the risk of declined renal function in East China.

LAP, which is determined by two components of WC and TG concentration, was first proposed by Kahn in 2005 to predict cardiovascular outcomes and mortality [12]. Epidemiologic studies further demonstrated that LAP could reflect visceral adiposity dysfunction and be associated strongly with dyslipidemia, insulin resistance, diabetes, PCOS and even metabolic syndrome [22–24]. In our study, we observed that, with the increase of the LAP quartiles, the eGFR level decreased and the prevalence of declined renal function increased. This significant trend was independent of age, sex, smoking, drinking, diabetes and hypertension, which implied that LAP may be a useful tool to identify the risk of declined renal function; we supplemented important evidence to the application of LAP.

Obesity and CKD are closely related, which is consistent with our findings. Chang and his colleagues discovered that the obese phenotype in metabolically healthy obese subjects could suffer from a higher risk for incident CKD [7], suggesting that obesity per se contributed to renal dysfunction and structural damage independent of metabolic abnormalities. In turn, the protective effect of weight loss on CKD is rather outstanding. Lifestyle intervention including caloric restriction and enhanced physical activity reduced the risk of CKD by 30% in overweight or obese diabetic patients compared to controls receiving an education intervention [25]. Weight reduction also helped improve obese CKD patients blood pressure, glomerular hyper-filtration and proteinuria [26]. Functional food often was added into regular pharmacological treatment to improve dyslipidemia and decrease the incidence of cardiovascular events, which was perhaps associated with the functional food’s anti-inflammatory effects [27]. These findings provided an option of weight reduction, functional food and ACE inhibition interventions in the treatment of overweight or obese patients with CKD [27, 28]. Athrough many findings were reported, the mechanism of how obesity initiated and exacerbated CKD was still not clarified. Recent studies revealed that renal compensatory hyper-filtration was likely to meet the enhanced metabolic demands of the excess weight. The continuous increase in intra-glomerular pressure would damage the kidney structure and increase the risk of advanced kidney disease [29, 30]. In an obese population, adipose tissue would result in release of inflammatory cytokines such as interleukin-6 or tumor necrosis factor-α to exacerbate kidney injury [31]. Visceral adipose tissue played key roles in the regulation of numerous adipokines and cytokines [32]. Clinical and animals studies demonstrated that ectopic lipid accumulation in the kidney contributed to obesity-related kidney injury [33–35]. Visceral adipose tissue was recognized as pathogenic fat deposition, and this deposition was associated more strongly with metabolic risk factors than the subcutaneous adipose tissue [36]. Our results supported those studies in that visceral adiposity dysfunction, which could be mirrored by LAP, was an independent risk factor for renal dysfunction.

There are several strengths in our study. First, the clinical meaning of this study is significant. Weight reduction helps alleviate the progression of CKD because obesity can be treated and controlled. Second, because the SPECT-China survey is a large population-based cross-sectional study, our results are more representative compared to a clinic-based population. Third, all the anthropometric measurements and biomedical analysis in this survey study were conducted by the same trained physician and students; thus, effective quality control was guaranteed. There are also some limitations in this study. First, because this is a cross-sectional study, we cannot determine a causal relationship between LAP and declined renal function. Second, the post-hoc analysis could be another limitation of this study. All the analyses between LAP and declined renal function were performed with existing data in an exploratory manner. Third, the most of participants enrolled in the study were Chinese residents, but LAP was firstly proposed and calculated based on white population. However, the application of LAP in a Chinese population had been reported in many diseases of insulin resistance, diabetes, NAFLD and even metabolic syndrome.

## Conclusions

Our results demonstrated that LAP was strongly associated with eGFR and the prevalence of declined renal function in East China. LAP could be a promising predictive index for the risk of declined renal function, which was independent of age, sex, smoking, drinking, diabetes and hypertension. Future research should focus on the underlying mechanism.

## Abbreviations

BAI: Body adiposity index
BMI: Body mass index
FBG: Fasting blood glucose
HbA1c: Glycated hemoglobin
HDL: High density lipoprotein
LAP: Lipid accumulation product
LDL: Low density lipoprotein
VAI: Visceral adiposity index
WC: Waist circumference
